# The Trade-Off of Virtual Reality Training for Dart Throwing: A Facilitation of Perceptual-Motor Learning With a Detriment to Performance

**DOI:** 10.3389/fspor.2020.00059

**Published:** 2020-05-21

**Authors:** Stefanie A. Drew, Madeline F. Awad, Jazlyn A. Armendariz, Bar Gabay, Isaiah J. Lachica, Jacob W. Hinkel-Lipsker

**Affiliations:** ^1^Visual Information Sciences and Neuroscience Laboratory, Department of Psychology, California State University, Northridge, CA, United States; ^2^Move-Learn Laboratory, Department of Kinesiology, California State University, Northridge, CA, United States

**Keywords:** virtual reality, ocular health, biomechanics, skill acquisition, sports, motor learning

## Abstract

Advancements in virtual reality (VR) technology now allow for the creation of highly immersive virtual environments and for systems to be commercially available at an affordable price. Despite increased availability, this access does not ensure that VR is appropriate for training for all motor skills. Before the implementation of VR for training sport-related skills takes place, it must first be established whether VR utilization is appropriate. To this end, it is crucial to better understand the mechanisms that drive learning in these new environments which will allow for optimization of VR to best facilitate transfer of learned skills to the real world. In this study we sought to examine how a skill acquired in VR compares to one acquired in the real world (RW), utilizing training to complete a dart-throwing task in either a virtual or real environment. We adopted a perceptual-motor approach in this study, employing measures of task performance (i.e., accuracy), as well as of perception (i.e., visual symptoms and oculomotor behavior) and motor behaviors (i.e., throwing kinematics and coordination). Critically, the VR-trained group performed significantly worse in terms of throwing accuracy compared to both the RW-trained group and their own baseline performance. In terms of perception, the VR-trained group reported greater acute visual symptoms compared to the RW-trained group, though oculomotor behaviors were largely the same across groups. In terms of motor behaviors, the VR-trained group exhibited different dart-throwing kinematics during training, but in the follow-up test adapted their throwing pattern to one similar to the RW-trained group. In total, VR training impaired real-world task performance, suggesting that virtual environments may offer different learning constraints compared to the real world. These results thus emphasize the need to better understand how some elements of virtual learning environments detract from transfer of an acquired sport skill to the real world. Additional work is warranted to further understand how perceptual-motor behaviors are acquired differently in virtual spaces.

## Introduction

Virtual reality (VR) systems have been under development for over 50 years, but the technology has only recently reached a point where it not only creates a high resolution, highly immersive experience, but is available on the commercial market at a relatively reasonable price (Slater and Sanchez-Vives, 2016; Arnaldi et al., 2018). Given these advancements, a wide range of industries are racing to embrace the new technology for training purposes before fully considering all implications. Integrations across the sporting domain are numerous and widespread (Düking et al., 2018), and recent work has examined its applicability to training skills such as soccer goalkeeping (Stinson and Bowman, 2014), rowing (Ruffaldi and Filippeschi, 2013), surfing (Farley et al., 2020), and marksmanship (Rao et al., 2018), to name a few. The expanding use of VR technology for acquisition or enhancement of sport skills has great promise, as a virtual world makes it possible to create training environments in which learning can take place that would otherwise be too costly, risky, or difficult to produce (Champney et al., 2014; Carruth, 2017). With these explorations, industries are becoming more interested in the basic science involved (Scarfe and Glennerster, 2015) and the physiological and psychological aspects of skill transfer from virtual to real-world environments still need to be thoroughly explored (Düking et al., 2018).

To date, there is some literature focused on the newest commercially available systems that demonstrates the promise of transfer of skills trained in virtual reality (Tirp et al., 2015). These reports have focused on single modalities during their examinations, such as task performance (e.g., Tirp et al., 2015), visual mechanisms (e.g., Mohamed Elias et al., 2019), or movement strategies (e.g., Nisky et al., 2014). Herein lies a critical weakness; Slater recently indicated that we should consider VR conceptually as a system that can alter a person's sensory input and thereby affect their motor output through effective environment design, and thus the perceptual and motor systems are not separable (Slater, 2014). To this end, a more comprehensive examination of VR in the context of both sensory input and motor output is warranted in order to better understand how to maximize sport skill acquisition in these environments.

The notion of the examination of a behavior in terms of perception and action is by no means a novel idea and has been investigated thoroughly across many disciplines (e.g., Gibson, 1979; Wagman and Blau, 2019). Over the years, researchers utilizing this approach have noticed that a change in perception ultimately affects resulting action (e.g., Stoffregen et al., 2004). Further, this approach has been carried over into the realm of virtual reality research where sensory stimuli typically differ from the real world, such as Gray's examination of training in a virtual environment for real-world baseball batting performance (Gray, 2017). However, the literature is sparse and more work is needed to examine skill acquisition across varying tasks and populations before a comprehensive understanding of perceptual motor learning in VR can be established.

Newell's Model of Constraints for skill acquisition is a well-established and widely accepted model (Newell, 1986) that describes how performance of a motor skill is determined by the interaction between perceptual and motor systems. In light of this model, a full understanding of skilled performance requires consideration of the underlying perceptual-motor mechanisms driving task performance. In turn, one's sensory perception of the environment and the way in which they coordinate movement are directly influenced by constraints related to the individual (e.g., the structure and function of body systems), the environment (e.g., characteristics of the physical performance space and sociocultural norms), and the task itself (e.g., rules or guidelines which dictate how the skill is performed). Therefore, it is not unreasonable to think that the individual, environmental, and task constraints offered by a virtual learning environment may differ from that of a real-world environment, thus idiosyncratically affecting task performance. In the context of Newell's Model, the different constraints imposed by a virtual learning environment would affect how that environment is perceived and, subsequently, how movement is coordinated to carry out a task. To truly examine virtual reality as an effective means for motor learning, one must consider how the combination of perceptual input and motor output of a learned task differs between virtual and real-world environments.

Previous work has also discussed how certain factors related to design of the virtual environment may either facilitate or detract from one's ability to transfer a learned skill to the real world. One factor, fidelity, refers to the degree to which a virtual environment resembles that of the real world (e.g., the resolution of graphics; Alexander et al., 2005; Bhargava et al., 2018). Another factor, immersion, describes aspects of the virtual environment that work to make the user feel more present in that world (as opposed to being in the real world; Rose and Chen, 2018; Slater, 2018). Some immersive elements of a virtual environment could include objects that have similar weight and shape as the same ones in the real world, or limiting outside sensory information coming from the real world (such as auditory stimuli). Therefore, when using a VR system for training of sports skills, it is important to maximize fidelity and immersion in order to ensure that environmental and task constraints overlap between environments as much as possible. However, the lay population does not often have access to the skills and resources required to design a custom environment, and thus may be reliant on commercially available platforms. All participants provided written informed consent, and all study protocols were approved by the university Institutional Review Board. It can be expected that there are discrepancies in environmental and task constraints between learning a new sport-specific skill using a commercially-available VR platform and performing that skill in the real world, possibly due to differences in fidelity and immersion. However, to date there is a lack of literature describing transfer of skills from VR to the real world using these environments, and whether the discrepancies between environments promote or detract from one's ability to acquire a new skill. Such work would help to better inform athletes and coaches of whether using a common VR platform for learning or enhancing sport skills is suitable, or if aspects of the environment such as fidelity or immersion need to be optimized to best facilitate transfer.

## Virtual Reality and Perceptual Input

In the natural world, two oculomotor systems work together to produce clear binocular viewing of an object. The accommodation system is responsible for keeping an object in focus by adjusting the focal power of the lens in the eye and the vergence system is responsible for maintaining binocular fixation on an object, with both eyes rotating in opposite directions to keep the object image on the fovea of each eye (Kim et al., 2014). In the real world, an object's distance to which the eyes must accommodate and converge are generally the same, so the demands to the vergence and accommodative systems are equal (Kim et al., 2014; Hoffman et al., 2015). However, modern technological advancements in stereoscopic displays and virtual reality provide novel environments in which these two systems no longer necessarily operate in synchrony. While wearing a VR head-mounted display (HMD), a user views a display that is positioned at a fixed location in front of their eyes, while focusing on a virtual object with varied binocular disparities and thus varied depths, creating a disassociation between accommodation and vergence cues (Kramida, 2015; Wilson and Soranzo, 2015). The resulting incongruity between these two ocular systems that occurs when viewing virtual reality displays has warranted some attention.

Discrepancies between the vergence and accommodation systems, also known as vergence-accommodation conflict (VAC), have been postulated by many to be a contributing factor of experienced visual discomfort when viewing stereoscopic displays (Wann and Mon-Williams, 2002; Emoto et al., 2005; Lambooij et al., 2009) and Hoffman et al. (2015) provide one of the first experimental demonstrations of the conflict causing visual fatigue and discomfort which have been extensively reported in the literature following stereoscopic display viewing. Investigation into these symptoms experienced by users led to the identification of a set of symptoms specific to VR experiences, coined “virtual reality-induced symptoms and effects” (VRISE; Cobb et al., 1999; Sharples et al., 2008). Symptoms reported after virtual reality usage include nausea, sickness, eyestrain, oculomotor effects, postural instability, and visual acuity (see also Nichols and Patel, 2002; Stoffregen et al., 2017). Ames et al. (2005) developed a symptom questionnaire to assess symptoms, the Virtual Reality Symptom Questionnaire (VRSQ), though this has not been applied to more advanced versions of VR technology.

Despite many advancements made in VR technology since the identification of VRISE, the symptoms experienced by VR users have continued to plague users of this new technology (Wilson and Soranzo, 2015). As previously reported, with the rapid increase in VR usage for training paradigms, it is critical that the impact and understanding of visual discomfort systems on the perceptual component of training be considered. Recently, Mohamed Elias et al. (2019) reported effects on oculomotor behaviors (observed increase in accommodative response and decrease in convergence) and symptoms after VR exposure; understanding these effects will help clarify impact on transfer. Their study provides a preliminary examination of oculomotor contributions to VR training, though the authors concluded that more examination is needed. Further, Mohamed Elias et al. (2019) focused on the oculomotor and task components of VR skill training but did not address the resulting kinematic outcomes involved, a critical piece that must be included to draw comprehensive conclusions about motor skill development in virtual environments.

## Virtual Reality and Acquisition of Kinematic Strategies

These changes in oculomotor behaviors, in the context of Newell's Model of Constraints, should have a direct effect on how movement is coordinated. While a wealth of literature exists describing how perception and action are coupled in the real world across a variety of motor skills and levels of expertise (Warren, 1990; Bertenthal et al., 1997; Kelso and Kay, 2016; Mallek et al., 2017), the way a virtual learning environment influences this relationship between sensorimotor body systems is largely unknown. While a multitude of studies report positive performance outcomes as a result of VR training (Adamovich et al., 2009), very few investigate the underlying kinematics of the performed movement and even fewer utilize a real-world-trained control group as a comparison of learning strategies. Of those that do, reports of positive kinematic transfer from virtual to real performance environments are mixed. For example, some recent work has cited similarities between virtual and real-world-trained individuals on kinematic movement strategies during a real-world performance test on sport-specific tasks such as handball goalkeeping (Bideau et al., 2004) and golf putting (Pataky and Lamb, 2018). On the other hand, another set of studies have described diverging real-world kinematic strategies between those trained in virtual and real environments on tasks such as reaching and grasping (Levin et al., 2015; Thomas et al., 2016). From these mixed results, it is apparent that multiple factors inherent in the virtual learning environment influence transfer of kinematic strategies from virtual to real worlds. For sport skills where utilization of the correct movement pattern is essential, it is important to investigate transfer at the kinematic level as a means to understand whether coordination strategies acquired in a virtual environment are applicable in the real world.

Further, beyond determining whether learning environments affect movement kinematics, it is also important to assess whether the kinematic strategies acquired during a learned skill actually promote successful completion of the task. Previous work has discussed that while individuals tend to demonstrate movement variability from trial-to-trial during performance of any motor task, those who are more proficient at a given skill can utilize that variability to their advantage through what is known as motor redundancy, as there are multiple movement patterns that can promote successful task performance (Cohen and Sternad, 2009). The coordination patterns that do not ultimately affect task performance are considered beneficial in that variance within this set of patterns allows for exploration of movement solutions—essentially, individuals are utilizing variance in a way that promotes learning (Cohen and Sternad, 2009; Sternad, 2018). Experts tend to make greater use of this set of movement solutions, previously termed the uncontrolled manifold (UCM), as they can explore new movement patterns without influencing task success (Scholz and Schöner, 1999). Conversely, novices tend to utilize movement variability outside of the UCM, indicating that they use coordination strategies that may detract from task performance (Rein et al., 2013; Nisky et al., 2014; Komar et al., 2015). Taking Newell's Model of Constraints into account, if the individual, environmental, and task constraints presented by a virtual learning environment differ greatly from that of the real world, the movement solutions within the UCM (or those that allow for exploration of strategies to find the optimal movement pattern through leaving task-related variables unaffected) may differ from virtual to real worlds—ultimately impacting task performance. To date, such an analysis has not been performed as a way to assess transfer of movement coordination from virtual to real worlds. Taken together with the perceptual elements of skill acquisition in VR, it is evident that a greater foundational understanding of how perceptual-motor systems are impacted by VR usage is needed to optimize how well-sport skills can transfer from virtual to real worlds.

## The Present Study

The purpose of this study was to determine how a single session of virtual reality training on a dart-throwing task, considered in the context of task performance and involved perceptual-motor mechanisms, compares to how those trained in the real world perform the skill. To this end, a secondary purpose was to evaluate how well a commonly-used virtual platform for dart throwing can be used in place of learning the skill in the real world, despite sub-optimal levels of fidelity and immersion. We formulated hypotheses for the (1) task performance as well as (2) the associated perception and (3) subsequent motor components of learning said task. As such, we hypothesized that overall, (1) those trained in VR would perform worse on a real-world follow-up test compared to those trained in the real world, as measured through a greater radial distance from the bullseye. We also anticipated that (2a) relative to real-world trained participants, virtual reality trained participants would experience a decrease in accommodative and vergence facility measures. Furthermore, we hypothesized that (2b) those trained in virtual reality would report greater number of visual discomfort symptoms post-training compared to those trained in the real world. Finally, we hypothesized that, compared to real-world-trained participants on a real-world follow-up test, VR-trained participants would exhibit (3a) different throwing arm joint angles at the time of dart release and (3b) have a lesser utilization of motor redundancy, as demonstrated through greater use of variability outside of the UCM.

## Methods

### Participants

Fifty participants were recruited to participate in this study through flyers and word-of-mouth. All participants provided written informed consent, and all study protocols were approved by the university Institutional Review Board. Eligibility criteria included consenting adults with a minimum age of 18, with normal or corrected to normal vision. Those with corrected to normal vision wore contacts during the study. Participants were screened via a questionnaire for conditions that cause visual discomfort (specifically; epilepsy, migraines, or head trauma), medications that can impact accommodative function (i.e., anti-anxiety agents, anti-arrhythmic agents, anticholinergics, and tricyclic anti-depressants) and participants with a history with any of these components were excluded from the study as they affect accommodative function. Participants were also screened for a history of epilepsy, seizures, loss of awareness, and for symptoms resembling an epileptic condition. Individuals with these conditions were excluded as VR systems may trigger seizures or other symptoms. In addition, individuals with high levels of dart-throwing experience (e.g., playing darts more than one time per month) or any chronic or acute upper-extremity injuries in the 6 months prior to enrollment in the study were excluded.

The data of nine participants were excluded due to poor stereopsis (see Instrumentation), administrative, or technological issues resulting in a total of 41 participants (22 male, 19 female) in the study. Participants were randomly assigned to either a virtual reality (VR) training condition or a real-world (RW) training condition. The VR group consisted of 22 (12 male, 10 female) participants while the RW group had 19 (10 male, 9 female).

### Instrumentation

Several standard optometric assessments were used prior to training. To assess stereoacuity (ability to detect differences in depth) and fine depth perception, participants completed the Circle Patterns of the standard Stereo Fly Test. Fixation disparity (misalignment of eyes when viewing with both eyes) was assessed with a Saladin Near Point Balance Card. Facility tests were performed to assess abilities of the accommodation and vergence systems to adjust to changing demands over time. Accommodation facility testing was performed with a +/– 2.00 D lens flipper over 2 min (and then calculated for 1 min duration). Vergence facility was measured with a flip prism 3 PD BI/12 PD BO over 2 min (and calculated for 1 min).

During the baseline, training, and follow-up trials three-dimensional marker coordinate data were recorded for the first five throws of each set at 150 Hz using an 8-camera motion capture system (Motion Analysis Corp., Santa Rosa, CA). 11 retro-reflective markers were placed on the following anatomical landmarks of participants' trunk and throwing arm: spinous processes of C7 and T10 vertebrae, jugular notch and xiphoid process of the sternum, left and right acromion processes, lateral and medial humeral epicondyles, radial and ulnar styloid processes, and the 3rd metacarpal of the hand.

For the dart-throwing task, a standard dartboard was set at regulation height and darts were used, participants were instructed to stand behind a line marking off a distance of 7 feet 9 ¼ inches (as what is listed as standard distance by the World Dart Federation) from the target. For participants assigned to train in a virtual environment, an HTC Vive CPO Education Edition (HTC, Taoyuan, TW) was used. The commercially available game, VR Darts Zone (Reality Busters Co, 2017), that simulates a bar with a standard dartboard available in it was pre-loaded onto the system. The VR space was calibrated as a 4 × 3 m rectangle using SteamVR (Valve, Bellevue, WA) and two lighthouses on opposing corners of the rectangle ~5 m above the floor tracked three-dimensional movement through the space.

There are a number of elements in this environment that reduced fidelity and immersion, and thereby affected the degree to which task and environmental constraints overlap between the virtual and real-world spaces. For one, in the real world the dart board was set to a single height for all participants regardless of their own height, while in VR the entire environment scaled to the height of the participants' HMD while it is being worn. This could affect the fidelity of the environment as dartboard heights are not consistent across learning environments. In addition, the participants in VR used a standard HTC hand controller, which involves holding and releasing a trigger instead of releasing a dart and has a different weight, distribution of weight, and shape than the standard real world dart. Moreover, the physics of flight and sense of gravity in the virtual environment may have also differed slightly from the real world. All of these factors may have decreased the levels of immersion in this virtual environment. As previously discussed, this environment was chosen as it was representative of a typical game that the lay population would have access to, where they do not have access to the resources or possess the technical skills to construct a virtual environment that perfectly replicates the real world.

Questionnaires were administered electronically via Qualtrics (Qualtrics, Provo, UT) on a Surface tablet device (Microsoft Corporation, Redmond, WA). These surveys included an acute symptom questionnaire administered before and after training (Drew et al., 2013), as well as the Virtual Reality Symptom Questionnaire (VRSQ) developed by Ames et al. (2005) and the Computer Usage Survey (Hayes et al., 2007) given after training. Participants also answered several demographic questions related to age, sex, height, and weight.

### Procedure

After giving informed consent, all participants were asked to complete a short, 4-question acute symptom questionnaire to determine baseline levels of acute symptoms experienced. Next, participants were seated in a side room and measures of stereoacuity, fixation disparity, accommodation facility, and vergence facility were measured. Following these optometric measurements, 11 retro-reflective markers were placed on the participants (see Instrumentation). Next, all participants completed a baseline set of dart throws where they were instructed to stand at the pre-marked location and to throw a set of five darts at their own pace. All throws were continuously recorded through motion capture. Once these throws were completed, researchers measured the radial distance of each dart's position from the center, recorded these distances as a measurement of accuracy, and removed the darts from the board. The participants were then instructed throw another set of five darts with the same data collection procedure repeated.

Participants assigned to the VR training group (*n* = 22) used the HTC Vive with the VR Darts game loaded. Participants were instructed on how to navigate the virtual room and told to spend the first 5 min exploring the virtual space, allowing them to acclimate to the new environment. Participants in the RW training group (*n* = 19) were similarly instructed to spend the first 5 min exploring the experimental space in the real world. After this familiarization period, all participants were instructed to begin throwing darts in their assigned environment for 25 min, over which they threw a total of 100 darts. In VR, throwing the dart involved squeezing a trigger to picking it up, and releasing the trigger during the throw at the point in time where the dart should be leaving the hand. After every set of 10 throws, all participants were instructed to rest for 1 min in order to minimize effects of physical strain. Participants continued in this manner until they had completed 100 dart throws. If participants completed all of their throws before the 25-min mark was reached, they were instructed to explore their environment (either virtual or real-world) for the remaining amount of time. This was done to ensure a consistent duration of VR exposure for the VR-training group and mirrored in the control group.

Following completion of the training session, accommodation facility and vergence facility were measured again for each participant in the same manner as the pre-training assessments. Participants were then instructed to return to the indicated place and to throw two sets of five darts in the same manner as described in the pre-training session, with motion capture data continuously recorded. Throughout baseline, training, and follow-up participants were not directly provided with any feedback in terms of throwing accuracy, though they were able to view the thrown dart's position on the board in both VR and the RW conditions. While the VR platform did provide information regarding their score, this does not correspond with radial distance from the bullseye. Next, participants completed several questionnaires administered via an iPad using Qualtrics software (Qualtrics, XM, Provo, UT). This included a post-training acute symptom survey, the Virtual Reality Symptom Questionnaire (VRSQ), the Computer Usage survey and several demographic questions.

### Data Analysis

All three-dimensional marker coordinate data were low-pass filtered using a 4th order Butterworth with a 6 Hz cutoff frequency and used to build a 4-segment model (trunk, upper arm, forearm, hand) in Visual 3D (C-Motion, Germantown, MD). This model was used to extract shoulder, elbow, and wrist joint angles during each throw. Next, using a custom MATLAB (MathWorks, Natick, MA) script, data were trimmed into individual throws. The start of each throw was identified as the time when the marker on the 3rd metacarpal on the throwing hand reached its maximum distance away from the target (i.e., was in a “cocked” position), while the end of the throw was marked as point in time where the angular radial-ulnar deviation velocity of the wrist joint reached a local minimum after achieving its peak angular velocity. Shoulder, elbow, and wrist joint angles were time-normalized from 0 to 100% of the throw and used to form ensemble curves for visualization of throwing kinematics before and after training. In addition, discrete shoulder, elbow, and wrist joint angles were extracted at the time of dart release—which we have termed “end joint configuration”—as a way to quantify whether kinematic strategies differed between groups. These discrete angles were binned into 14 sets of 5 throws: 2 before training (Pre 1 and Pre 2), 10 during training (the first 5 throws in each of the 10 sets of 10 throws, Training 1, Training 2…Training 10), and 2 after training (Post 1 and Post 2).

Additionally, we also sought to determine how participants in each group coordinated motion of the joints of the throwing limb relative to a task-related variable using an uncontrolled manifold (UCM) approach. For this study, we computed a three-dimensional vector from the throwing arm shoulder joint center to the marker on the 3rd metacarpal on the throwing hand. This vector was used as the task-related variable that we anticipated the dart thrower needed to stabilize to promote successful throwing performance, as previous work has shown that variability of hand position at the time of dart release is reduced following practice (Smeets et al., 2002). While the following will briefly outline how the UCM is computed, a more detailed methodology can be found in other works (Scholz and Schöner, 1999; Tseng et al., 2002; Domkin et al., 2005). While the UCM approach has not been used to analyze joint-level coordination for dart-throwing, it has been used to examine similar tasks such as manual tool use (Rein et al., 2013) and Frisbee throwing (Yang and Scholz, 2005).

To determine how changes in arm joint angles affected the throwing arm's hand position at different time points of the throw, we developed a forward kinematic model relating throwing arm segmental lengths and joint angles to the task-related variable. This relationship is represented by a 3x4 Jacobian matrix containing partial derivatives relating small changes in joint angles (degrees of freedom (DF) = 4: shoulder flexion/extension, shoulder ab/adduction, elbow flexion/extension, wrist radial/ulnar deviation) to the three-dimensional vector coordinates of the task-related variable (*n* = 3). The null space of this Jacobian matrix is therefore representative of the joint configurations that do not affect the task-related variable, and the deviations of joint angles from the reference configuration (the mean of each joint angle for each of the 14 sets of 5 throws, 14 total reference configurations) can be projected onto the null space. This component of the deviations from the reference configuration are hence within the UCM (deviations that do not affect the task-related variable), and the orthogonal component is representative of deviations that do affect the task-related variable. From both components, the variances per degree of freedom are then computed, one representative of joint angle variance that is within the UCM (goal equivalent variability; GEV) and the other representative of variance orthogonal to the UCM (non-goal equivalent variability; NGEV).

The normalized difference between GEV and NGEV—termed the index of motor abundance (IMA; Tseng and Scholz, 2005; Auyang et al., 2009) can therefore represent how participants are utilizing variance in joint configurations. An IMA closer to 1 is indicative of much more utilization of GEV than NGEV, therefore revealing that participants coordinated joint angle variability to explore different movement solutions without changing the task variable. On the contrary, an IMA closer to −1 indicates more NGEV than GEV, meaning that participants utilized variance in joint configurations that changed the position of the task-related variable. Finally, an IMA close to 0 indicates no coordination strategy was used. We performed this analysis at five different time slices in the throw: 0% (beginning of dart throw), 25, 50, 75, and 100% (time of dart release). In the context of our study, more skilled dart-throwers should utilize variance to their advantage (i.e., greater GEV than NGEV or an IMA closer to 1), which would imply that they were exploring different throwing motions that allowed for hand position relative to the shoulder to remain relatively constant throughout the throw. Conversely, less skilled throwers should have a lesser IMA than skilled throwers, with the implication being that they utilized more variance in joint configurations that changed the position of the hand. As such, this analysis allows for a more in-depth look beyond discrete kinematic measures at how the throwing motion is coordinated with relation to successful throwing performance, and can help to further understand any differences between practice groups in terms of dart-throwing performance or joint-level kinematics.

### Statistical Analyses

To compare throwing performance of each group (hypothesis 1), accuracy on the dart-throwing task was calculated before and after training for both groups. The accuracy measurements were taken by two researchers and these values were averaged between the two to ensure a more robust measurement of accuracy for each throw. Average accuracy was calculated across the first five throws and across the second five throws, and an average accuracy score calculated from these two values. To test for the main effects of practice group and time on accuracy, we used a two-factor rANOVA (α = 0.05).

Prior to performing any of the aforementioned rANOVAs, we tested for assumptions of homogeneity of variance (Levene's Test of Homogeneity of Variance), homogeneity of covariance (Box's Test of Equality of Covariance), sphericity, and normality (Shapiro-Wilk's test). Greenhouse-Geiser corrections were used when sphericity was violated, and we made square root transformations or ran non-parametric tests if the assumption of normality was violated. When a rANOVA was revealed to be statistically significant, we conducted follow-up Bonferroni-corrected pairwise comparisons.

To address hypothesis 2a, two two-factor repeated-measures analyses of variance (rANOVAs, α = 0.05) were performed, testing for the main effects of practice group and time on (1) accommodation facility and (2) vergence facility. Two time points were included, one before training and one after. Similarly, in pursuit of hypothesis 2b, multiple statistical tests were performed to test for the main effects of practice group and time on composite scores for the three symptom questionnaires (acute symptom survey, VRSQ, CUS). First, composite scores for the acute symptom survey were calculated and categorized into one of two possible categories: No Symptoms Reported or Symptomatic. This logic for this organization of the data was 2-fold: (1) the response scale for each of the four questions slightly differed and (2) the scores violated Levene's test of homogeneity. Therefore, binomial proportions were established for both the virtual reality training and the real-world training groups and chi-square tests of homogeneity performed. Next, exploratory factor analysis (EFA) of the VRSQ has found a clear two-factor solution, with factors of General Body Discomfort and Eye Related Symptoms onto which all but one of the questions loaded, therefore composite scores for these two factors were calculated (Del Cid et al., submitted) for further analysis. A Mann-Whitney U test was performed to examine differences between the two training groups on the Eye Related Symptoms and General Body Discomfort factors. Finally, previously performed EFA on the CUS has revealed a four-factor solution onto which the 20 of the 21 survey questions mapped. These factors were Visual Discomfort, Back Discomfort, Vision Difficulties and Extremity Discomfort (Del Cid et al., submitted). Composite scores for each of these factors were calculated and Mann-Whitney U tests were performed for each factor of Visual Discomfort, Back Discomfort, Vision Difficulties, and Extremity Discomfort.

To test for the main effects of practice group and time on throwing arm shoulder, elbow, and wrist joint angles (hypothesis 3a), we performed three repeated-measures, two-factor analyses of variance (rANOVAs; α =0.05). Fourteen time points were included: two bins of five throws before training, ten during training, and two after training. Similarly, to address hypothesis 3b we used five rANOVAs (α =0.05) to test for the main effects of practice group and time on the five IMA time slices (0, 25, 50, 75, 100%), with two time points included—one before training (average IMA for each of the two bins of 5 throws) and one after.

## Results

### Task Performance

**Figure 1:**
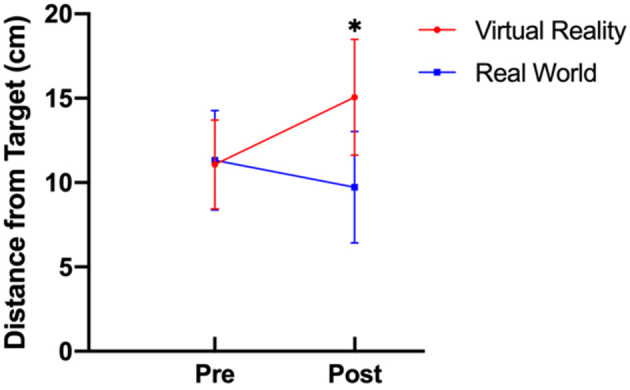
Dart-throwing accuracy before and after training for the virtual reality and real-world training groups (* denotes *p* < 0.05).

There was a statistically significant interaction between training group and time on dart throw accuracy [*F*_(1, 39)_ = 35.48, *p* < 0.001, ηp^2^ = 0.476]. Univariate analyses examining the differences between groups revealed that before training, dart throw accuracy was not significantly different in the virtual reality training group compared to the real-world training group (*p* > 0.05). Following training, the accuracy of the virtual reality training group was significantly worse (e.g., further radial distance from the bullseye) than the real-world training group [*F*_(1, 39)_ = 25.627, *p* < 0.001, ηp^2^ = 0.397]. There was a statistically significant effect of time on accuracy for the VR training group [*F*_(1, 21)_ = 37.88, *p* < 0.001 ηp^2^ = 0.643] such that participants performed less accurately after training (i.e., further radial distances from the bullseye). There was also a statistically significant effect of time on accuracy for the RW training group [*F*_(1, 18)_ = 5.61, *p* =0.029, ηp^2^ = 0.238], such that participants were more accurate (i.e., closer radial distances to the bullseye) after training (Figure 1).

### Perceptual Measures

**Figure 2:**
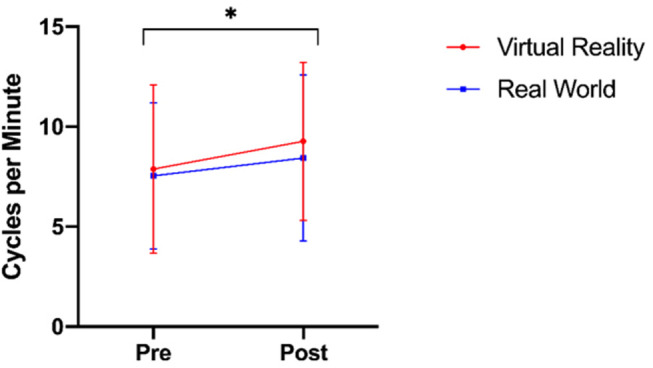
Accommodation facilities before and after training in both virtual-reality and real-world training groups (* denotes *p* < 0.05).

**Figure 3:**
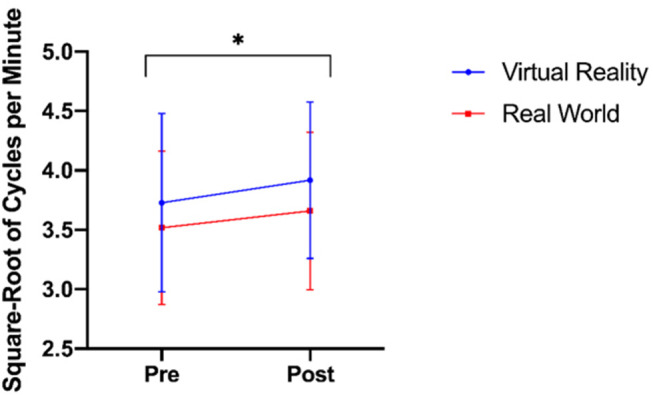
Square-root transformed vergence facilities before and after training (* denotes *p* < 0.05).

There was no significant interaction between time and group on accommodation facility [*F*_(1, 39)_ = 0.487, *p* = 0.489, ηp^2^ = 0.012]. The main effect of time showed significantly different accommodative facilities before and after training [*F*_(1, 39)_ = 10.119, *p* < 0.05, ηp^2^ = 0.206]. There was no significant main effect of training group [*F*_(1, 39)_ = 0.238, *p* =0.628, ηp^2^ = 0.006; Figure 2]. The pre-training vergence facility test was not normally distributed as assessed by Shapiro-Wilk's test (*p* < 0.05), nor was the post-training vergence facility test (*p* < 0.05), therefore a square-root transformation was performed on the data, resulting in a normal distribution (*p* > 0.05). There was no significant interaction between time and group on vergence facility [*F*_(1, 39)_ = 0.239, *p* = 0.628, ηp^2^ = 0.006]. The main effect of time showed significantly different vergence facilities before and after training [*F*_(1, 39)_ = 12.174, *p* = 0.001, ηp^2^ = 0.238]. There was no significant main effect of training group [*F*_(1, 39)_ = 1.278, *p* = 0.265, ηp^2^ = 0.032; Figure 3].

**Figure 4:**
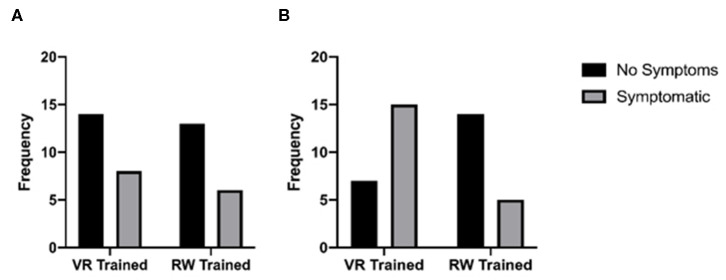
Frequency distributions of symptom reporting **(A)** before training and **(B)** after training.

In the virtual reality training group, 8 of the 22 participants (36.4%) reported experiencing acute symptoms, while in the real-world training condition, 6 of the 19 participants (31.6%) reported experiencing symptoms prior to training. The difference in proportions of 0.048 was not statistically significant (*p* = 0.747). Following training, in the virtual reality training group, 15 participants (68.2%) reported symptoms while in the real-world training group, 5 participants (26.3%) reported symptoms, a statistically significant difference in proportion of 0.419 (*p* = 0.007) (Figure 4). Distributions of scores for the VRSQ were similar for the two training groups, as assessed by visual inspection. No statistically significant differences were found between groups for either factor (General Body Discomfort or Eye-Related Symptoms; *p* > 0.05). Distributions of scores for each factor in the CUS were similar, as assessed by visual inspection, and no significant difference was found between the scores of each factor (Visual Discomfort, Back Discomfort, Vision Difficulties and Extremity Discomfort; *p* > 0.05).

### Action Measures

**Figure 5:**
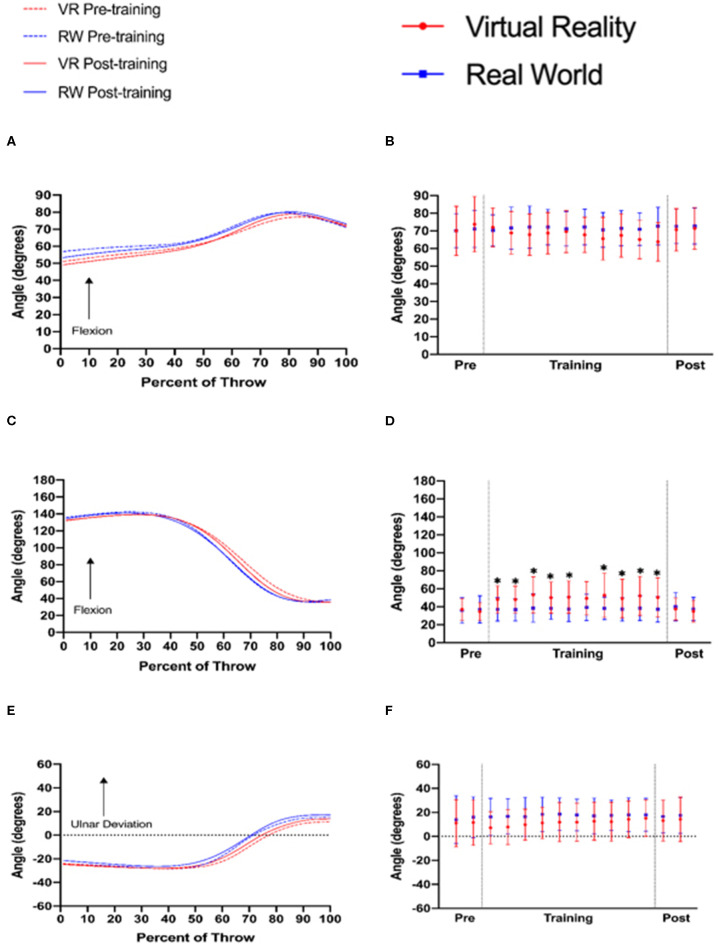
Ensemble curves representing mean joint excursions from start (0%) to finish (100%) of the dart throw (left column) and discrete joint angles (right column) at the time of dart release for participants in the virtual reality and real-world practice groups before (pre) and after (post) training. **(A,B)**—Shoulder flexion/extension, **(C,D)**—elbow flexion/extension, and **(E,F)**—wrist radial/ulnar deviation (* denotes *p* < 0.05).

A visual examination of shoulder, elbow, and wrist joint ensemble angles qualitatively demonstrated that there were no differences in throwing kinematics between training groups before or after training (Figures 5A,C,E). The lack of qualitative differences in joint angles before and after training is corroborated by statistical analyses. For the end shoulder joint configuration, there was no significant main effect of time [*F*_(13, 39)_ = 1.843, *p* = 0.131, ηp^2^ = 0.047] or for the time × group interaction [*F*_(13, 39)_ = 2.192, *p* =0.08, ηp^2^ = 0.056; Figure 5B]. For the elbow, there was a significant main effect of time [*F*_(13, 39)_ = 6.094, *p* < 0.001, ηp^2^ = 0.141] and a significant time × group interaction [*F*_(13, 39)_ = 5.539, *p* < 0.001, ηp^2^ = 0.130]. Follow-up Bonferroni comparisons reveal that these differences lie solely during training, as the VR training group released the dart with significantly greater elbow flexion compared to the control group for training times 1–5 and 7–10 (*p* < 0.05; Figure 5D). There were no significant differences before or after training, however. Finally, for the wrist joint there was no significant main effect of time [*F*_(13, 39)_ = 2.604, *p* = 0.057, ηp^2^ = 0.066] or a significant time × group interaction [*F*_(13, 39)_ = 1.226, *p* =0.304, ηp^2^ = 0.032; Figure 5F].

**Figure 6:**
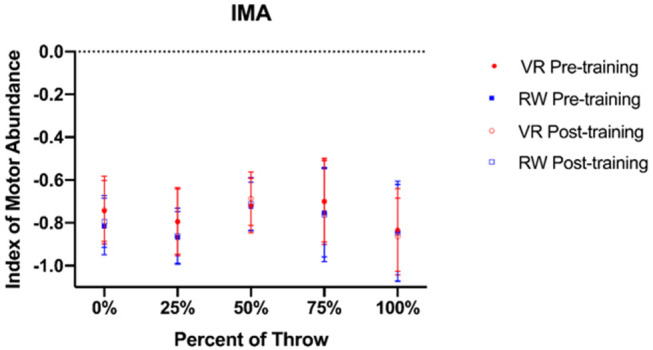
Index of Motor Abundance (IMA) for those trained in virtual reality and the real world before (pre) and after (post) training.

All ANOVAs demonstrated no significant main effect of time [0%—*F*_(1, 39)_ = 1.725, *p* = 0.197, ηp^2^ = 0.045; 25%–*F*_(1, 39)_ = 0.092, *p* = 0.764, ηp^2^ = 0.002; 50%–*F*_(1, 39)_ = 1.771, *p* = 0.191, ηp^2^ = 0.046; 75%–*F*_(1, 39)_ = 0.036, *p* = 0.850, ηp^2^ = 0.001; 100%–*F*_(1, 39)_ = 1.4127, *p* = 0.242, ηp^2^ = 0.03] or a time × group interaction [0%–*F*_(1, 39)_ = 0.723, *p* = 0.401, ηp^2^ = 0.019; 25%–*F*_(1, 39)_ = 0.120, *p* = 0.731, ηp^2^ = 0.003; 50%–*F*_(1, 39)_ = 0.326, *p* = 0.572, ηp^2^ = 0.009; 75%–*F*_(1, 39)_ = 0.023, *p* = 0.880, ηp^2^ = 0.001; 100%–*F*_(1, 39)_ = 0.528, *p* = 0.109, ηp^2^ = 0.014] on Indices of Motor Abundance (Figure 6).

### Throwing Velocity

**Figure 7:**
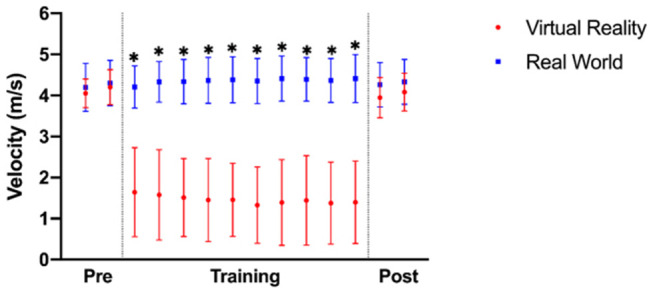
Peak dart-throwing velocity for participants trained in virtual reality (VR) and the real-world (RW) before (Pre), during (Training), and after (Post) training (* denotes *p* < 0.05).

After addressing all hypotheses, we performed an additional *post hoc* analysis to further determine the motor mechanism behind the noted group differences in task performance following training. Since previous work has detailed how experts throw darts at a higher velocity than novices (Schorer et al., 2012), we anticipated that the VR training group would throw at a significantly lower velocity following training compared to the real-world group, thereby impacting their throwing accuracy. To pursue this, we computed the peak resultant linear velocity of the marker placed on the 3rd metacarpal of the throwing hand from start to finish of the throw for both groups at the 14 measured time points (2 before training, 10 during training, 2 after training). This analysis revealed a significant main effect of time [*F*_(13, 39)_ = 90.076, *p* < 0.001, η2 = 0.709] and a significant time × group interaction [*F*_(13, 39)_ = 102.834, *p* < 0.001, η^2^ = 0.735]. Follow-up Bonferroni-corrected pairwise comparisons between groups at each time point indicate that the VR training group threw with a significantly lower velocity for all 10 time points during training (*p* < 0.001). However, at the two time points after training there were no significant differences between groups (Post-training 1—*t*_(1, 39)_ = −1.892, *p* = 0.066; Post-training 2—*t*_(1, 39)_ = −1.515, *p* = 0.138; Figure 7).

## Discussion

The purpose of this study was to examine the effects of a training session held in virtual reality compared to one held in the real world on a dart-throwing task.

While the VR group seemed to acquire similar perception and action strategies as the real-world group, these strategies did not translate into effective performance in the real world. Moreover, training in VR seemed to be detrimental as this group had significantly worse accuracy compared to how they performed prior to training. These results support hypothesis 1 and indicate that virtual training had a detrimental effect on real-world performance. Because Newell's Model of Constraints describes how performance of skill is dictated by the interaction between perception and action (Newell, 1986), we utilized multiple perceptual-motor measures as an attempt to uncover the mechanisms behind virtual skill acquisition. However, the lack of perceptual-motor differences between training groups on the real-world follow-up tests indicate that participants were behaving similarly in terms of perception and action. Thus, it is possible that some components of fidelity, such as the dartboard height being scaled to the participant in the virtual environment instead of being standard across all participants, may have negatively affected accuracy. To speculate, participants may have acquired a similar dart-throwing form to those in the real world, but were unable to correct that form when adapting their strategy to a slightly different dart board height. Further research should investigate whether scaling a virtual environment to better replicate the real world will in turn impact transfer of learned sport skills.

We hypothesized that (2a) virtual-reality trained participants would experience a decrease in accommodative and vergence facilities compared to real-world trained participants. This hypothesis was not supported by the data, as no group differences were observed, but both groups showed a significant increase in both facilities over time. These data are inconsistent with reports of Mohamed Elias et al. (2019), who reported an increase in accommodation response and a decrease in vergence. There are several possible explanations for this discrepancy between results. One possibility could be virtual reality task differences, as Mohamed Elias et al. (2019) utilized a virtual reality game that simulates continuous motion with content varying in distance on the virtual plane, our task involved remaining relatively stationary while focusing on a far target. As these two tasks had differing oculomotor demands, perhaps it is unsurprising that there were differences observed in the oculomotor behaviors. Furthermore, the oculomotor assessment measures themselves differed between these two studies; we employed accommodative and vergence facilities tests while Mohamed Elias et al. (2019) measured accommodative response and vergence stability. While our differing measures have allowed both Mohamed Elias et al. (2019) and our team to draw general conclusions about accommodative and vergence behaviors, it is likely that differences in the measurements made could account for some of the conflicting results. Arnaldi et al. (2018) have also suggested that while the vergence-accommodation conflict is a problem, perhaps a bigger issue is rapid changes of vergence demands, as reported by Emoto et al. (2005). This assertion would explain discrepancies between the findings of Mohamed Elias et al. (2019) and our own, as their target had changes in the visual plane while ours was at a fixed position; based on these differences, one might expect greater vergence problems reported with the target that varies in depths, as was observed.

Hypothesis 2b, which postulated that participants in the virtual reality training condition would report a greater number of symptoms post-training, was partially supported. There was a significantly greater proportion of symptoms reported on the acute symptom survey, but no significant differences were found between composite scores for the VRSQ or the CUS symptom surveys. The finding that the virtual reality training group experienced greater symptoms is consistent with extensive literature reporting symptoms after VR use (Nichols and Patel, 2002), including reports after using more advanced technology (Wilson and Soranzo, 2015; Mohamed Elias et al., 2019). The lack of significant results on the VRSQ and CUS may have several explanations. One possibility is that these surveys were administered after post-training accommodation and vergence facilities were measured and the final real-world dart-throwing assessment were made, a time of at least 5 min, and symptoms surveys administered subsequently after final dart throw measurements. As literature has reported that some symptoms experienced have a very short duration before being resolved (Mon-Williams et al., 1993; Rushton et al., 1994; Ames et al., 2005), it is possible that symptoms experienced may diminish to a point where subjective reporting did not detect differences; the broad acute symptom survey was administered first, and consisted of only four questions, while it was followed by the 14-question VRSQ and 21-question CUS; the length of these surveys may have resulted in additional time for symptoms to resolve. Furthermore, as with any survey, it is possible that participants were less engaged with the task of reporting for the lengthy 35 questions of the combine VRSQ and CUS. Future studies may want to consider selecting a single measure with a reduced number of items that require less time to complete.

We had also hypothesized that (3a) following training, participants in the virtual reality training group would throw with significantly different arm joint angles in the end configuration compared to real-world-trained participants. The results of this study do not support this hypothesis, as during the real-world follow-up tests there were no differences between groups for shoulder, elbow, or wrist joint angles in the end configuration. These results are surprising, given that during training the virtual reality training group exhibited significantly greater elbow flexion angles at the point of dart release during training, indicating that they were not extending their arm at the elbow as far as those trained in the real-world. This difference could be due to the fact that during VR training, participants had to hold a controller and release a trigger to throw a dart as opposed to throwing an actual dart. Previous work has discussed how certain physical parameters of objects influence individuals' reaching and grasping behavior (Johansson and Cole, 1992), and therefore the difference in how the virtual dart is manipulated compared to holding a real dart may serve as a task constraint. In turn, throwing kinematics (the motor component of Newell's Model of Constraints) were affected. Despite this unique throwing pattern, those trained in VR were able to rapidly adapt their throwing motion to the real world, as their throwing motion mirrored that of the real-world-trained participants on the follow-up test. Hence, the unique throwing behavior imposed by the constraints of this virtual learning environment does not transfer to the real world, which could indicate that manipulated objects may not need to be fully immersive in terms of weight, weight distribution, shape, and grip orientation if the acquisition of a specific kinematic pattern is the only goal of a virtual learning of sport skills. These results support previous work that has described how individuals who have learned a skill in virtual reality utilize similar kinematic patterns compared to those who learned the skill in the real world (Bideau et al., 2004; Viau et al., 2004; Fluet et al., 2015; Parijat et al., 2015; Pataky and Lamb, 2018). Overall, it seems that use of a standard handheld VR controller in place of a real-world object may be suitable for acquisition of kinematic patterns despite reducing immersion, although more work is needed to ensure that this observation holds true across different skills and sport performance contexts.

This lower level of immersion imposed by the use of a controller instead of a dart in VR and the increased weight of the controller, in combination with lower-fidelity aspects of the virtual environment (e.g., differences in dart projectile motion in VR compared to the real world), may be also be a reason why throwing velocity in VR was slower. However, since participants in the VR group threw at a velocity comparable to the real-world group on the real-world follow-up test, it appears that the VR group acquired a kinematic throwing pattern that is adaptable to different performance contexts. This observation is further supported through the UCM analysis, where participants in both groups utilized movement variability in the same way on the follow-up test, as there was no difference in IMA at 0, 25, 50, 75, or 100% of the throw. These results do not support hypothesis 3b, which predicted that the virtual reality training group would demonstrate lesser utilization of goal equivalent variability on the real-world follow-up test, as evidenced by an IMA closer to −1. Like the discrete joint angles, these results are also surprising given the relatively lower levels of fidelity and immersion in the virtual environment. Interestingly, both groups also exhibited an IMA that was <0 for all time points in the throw, indicating that all participants were utilizing more joint angle variability that affected the task-related variable (NGEV) than variability that did not (GEV). These results may have occurred for one of two reasons. First, the task-related variable (throwing hand position relative to the shoulder) may not be important for an individual to stabilize during a dart throw, and therefore individuals can vary its position while still effectively carrying out the throw. Other work has described how dart-throwing accuracy can be related to other variables such as release point, the angle of the dart, and release velocity (Kudo et al., 2000). Therefore, individuals may be able to compensate for a changing hand position by altering some or all of the other control variables, and thus hand position at the time of dart release may not be an important control variable. Second, as participants only underwent one acute session of training, it is possible that all individuals did not become proficient to the point where they were able to minimize variability of the task-related variable. Third, it should be noted that cognitive processes such as attention may also have had some effect on throwing coordination (Lohse et al., 2014; Sherwood et al., 2014). Regardless of the reason why participants organized variance, these results are noteworthy given that those who trained in VR acquired similar coordination patterns to those in the real world, perhaps lending further credence to the thought that the virtual environment does not need to be perfectly optimized in order to facilitate transfer of motor strategies.

There are other aspects of perception which should be considered by future work to provide a higher-resolution analysis of how performance can be negatively impacted by VR training. For one, the acute symptoms experienced by VR users may have been distracting to the participant or may have influenced their gaze behavior. Research has described how visual symptoms influence gaze behavior during performance of various tasks (van Leeuwen et al., 1999), but greater understanding of the relationship between these symptoms and actual gaze behavior specifically as a result of VR usage would be beneficial to understanding how perception is altered by this medium. A wealth of literature has described how, for far aiming tasks such as darts, experts utilize a “quiet eye,” where the eye is stabilized and fixated on a target for a longer duration than novices (e.g., Vickers, 1996; Oppici et al., 2017). Hence, the reported usage symptoms may not have allowed for VR-trained participants to effectively stabilize their gaze during practice. Future work should utilize eye-gaze tracking measures in order to further determine how the use of an HMD for VR usage may affect visual perception of the task.

There are also additional measures of dart-throwing motor behavior that could help to explain performance differences in the future. For example, because VR-trained participants had to use a controller with a trigger to throw the virtual darts, and the controller was heavier (470 g controller vs. 18–24 g darts), their level of immersion in the virtual environment may have been affected. Immersion, an objective factor of a VR system that allows for a person to believe they are present in a virtual space (Slater, 2018), has been previously demonstrated to impact transfer of skills to the real world (Alexander et al., 2005). Future studies investigating VR to real-world transfer for throwing tasks should examine how changing the objects being manipulated in a virtual environment impact transfer of motor behavior and should utilize other projectile motion measures in order to further explain any performance differences compared to those trained in the real world.

## Limitations

In addition to those already described specifically relating to each hypothesis, several additional limitations of our study should be noted. While our design included a real-world training group as a control group to be compared to the virtual reality trained group, perhaps additional information could be gleaned from the inclusion of a third group that would not undergo any training at all. As the target task performance following training declined in the virtual reality training group but improved in the real-world training group, the inclusion of this third group would allow additional comparisons to be made between each training group and no training group at all. A second limitation, as discussed previously, is that several aspects of VR fidelity and immersion (e.g., dartboard height scaling and controller usage) may have limited the degree to which participants in VR were able to transfer their skill to the real world. However, this virtual environment was specifically was chosen as it is free for use and therefore may reflect the quality of common platforms used for training of other sport skills. Additionally, some participants may have had slightly more dart-throwing expertise than others prior to training (though none had extensive experience). This moderate familiarity may have had some impact on the learning curve of those participants on the task. A final limitation to our study was that vergence facilities were always performed after accommodative facilities; it is possible that during the accommodative facility tests, participants' vergence system is able to sufficiently adapt to the real world after VR exposure such that any potential dysfunction could be ameliorated in time for vergence assessment. Future work would benefit from a counterbalancing of measures to avoid this potential confound.

## Conclusions

In summary, the most note-worthy finding is that the virtual reality group performed significantly worse on the throwing task compared to their baseline, while the real-world group improved in performance. This outcome occurred despite the lack of differences between oculomotor behaviors and real-world task throwing strategies; we found that following training, those trained on a task in virtual reality demonstrated greater acute visual symptoms but similar oculomotor behaviors as those trained in the real world. In addition, during training, those in VR utilized a unique kinematic throwing strategy compared to the real-world group as evidenced by less elbow extension during the throw and a slower throwing velocity. However, following training, they adopted throwing strategies that mirrored those of the real-world group, perhaps demonstrating a rapid adaptability of coordinated movement between virtual and real environments. These results are noteworthy given the lower levels of fidelity and immersion of the virtual environment, which thereby limited the amount of overlapping environmental and task constraints between performance contexts. Thus, VR training platforms may not require optimization of fidelity and immersion to mirror real-world training, if acquisition of specific kinematic patterns for sport skills are a training goal. Future work should systematically manipulate aspects of fidelity and immersion in virtual environments to further clarify these results. Thus, it appears that other perceptual-motor factors or design factors may be present that detract from one's ability to transfer skills from virtual to real worlds. Future work should examine these performance differences across other sport skills and using other perceptual-motor measures to further generalize these results. In total, it is critical that those studying acquisition of sport skills in VR adopt an interdisciplinary approach to examining the underlying mechanisms of learning. A better understanding of human interactions with virtual environments will inform athletes, coaches, and the scientific community as to how to best implement a virtual training paradigm for acquisition of sport skills and how to better optimize virtual learning environments to maximize that acquisition. Given the exponential growth in VR utilization across the sporting domain, it is more critical than ever in both research and industry to consider the multiple dimensions through which VR usage can impact users.

## Data Availability Statement

The datasets generated for this study are available on request to the corresponding author.

## Ethics Statement

The studies involving human participants were reviewed and approved by Institutional Review Board California State University, Northridge. The patients/participants provided their written informed consent to participate in this study. This study adhered to the principles of the Declaration of Helsinki.

## Author Contributions

SD, JH-L, MA, and IL contributed to the conception and design of the study. MA, JA, and IL were responsible for all data acquisition. IL completed all data post-processing. SD, JH-L, IL, and BG performed statistical analyses. SD and JH-L wrote the manuscript. All authors contributed to manuscript revision, read and approved the submitted version.

## Conflict of Interest

The authors declare that the research was conducted in the absence of any commercial or financial relationships that could be construed as a potential conflict of interest.
